# Unlocking Precision Medicine for Prognosis of Chronic Kidney Disease Using Machine Learning

**DOI:** 10.3390/diagnostics13193151

**Published:** 2023-10-08

**Authors:** Yogita Dubey, Pranav Mange, Yash Barapatre, Bhargav Sable, Prachi Palsodkar, Roshan Umate

**Affiliations:** 1 Department of Electronics and Telecommunication Engineering, Yeshwantrao Chavan College of Engineering, Nagpur 441110, India; yogeetakdubey@yahoo.co.in (Y.D.); pranavmange2015@gmail.com (P.M.); yashbarapatre2406@gmail.com (Y.B.); bhargavsable2002@gmail.com (B.S.); 2 Department of Electronics Engineering, Yeshwantrao Chavan College of Engineering, Nagpur 441110, India; prachi.palsodkar@gmail.com; 3 Department of Research and Development, Jawaharlal Nehru Medical College, Datta Meghe Institute of Higher Education and Research, Sawangi, Wardha 442001, India

**Keywords:** chronic kidney disease (CKD), prognosis, machine learning (ML), gradient boost (GB), decision tree (DT), K-nearest neighbors (KNN), random forest (RF), histogram boost (HB), XGBoost (XGB)

## Abstract

Chronic kidney disease (CKD) is a significant global health challenge that requires timely detection and accurate prognosis for effective treatment and management. The application of machine learning (ML) algorithms for CKD detection and prediction holds promising potential for improving patient outcomes. By incorporating key features which contribute to CKD, these algorithms enhance our ability to identify high-risk individuals and initiate timely interventions. This research highlights the importance of leveraging machine learning techniques to augment existing medical knowledge and improve the identification and management of kidney disease. In this paper, we explore the utilization of diverse ML algorithms, including gradient boost (GB), decision tree (DT), K-nearest neighbor (KNN), random forest (RF), histogram boost (HB), and XGBoost (XGB) to detect and predict chronic kidney disease (CKD). The aim is to improve early detection and prognosis, enhancing patient outcomes and reducing the burden on healthcare systems. We evaluated the performance of the ML algorithms using key metrics like accuracy, precision, recall, and F1 score. Additionally, we conducted feature significance analysis to identify the most influential characteristics in the detection and prediction of kidney disease. The dataset used for training and evaluation contained various clinical and demographic attributes of patients, including serum creatinine level, blood pressure, and age, among others. The proficiency analysis of the ML algorithms revealed consistent predictors across all models, with serum creatinine level, blood pressure, and age emerging as particularly effective in identifying individuals at risk of kidney disease. These findings align with established medical knowledge and emphasize the pivotal role of these attributes in early detection and prognosis. In conclusion, our study demonstrates the effectiveness of diverse machine learning algorithms in detecting and predicting kidney disease. The identification of influential predictors, such as serum creatinine level, blood pressure, and age, underscores their significance in early detection and prognosis. By leveraging machine learning techniques, we can enhance the accuracy and efficiency of kidney disease diagnosis and treatment, ultimately improving patient outcomes and healthcare system effectiveness.

## 1. Introduction

A breakthrough in the field of chronic kidney disease (CKD) prediction was caused by the development of machine learning (ML) algorithms [1]. Recent strategies have been demonstrated to offer the best gradient support potential to achieve 96% accuracy in CKD outcome prediction [2]. Many studies showing the effectiveness of various algorithms, including decision trees (DT), K-nearest neighbors (KNN), random forests (RF), histogram boosting (HB), XGBoost (XGB), and support vector machines (SVM), have been motivated by this performance. The versatile algorithmic selection process plays a crucial part in the attractive field of CKD prediction. Guided by factors such as data availability, computing resources, and the right targets, researchers have used a clever strategy by deploying various algorithms. These algorithms are subjected to performance evaluations compared to tests on the same data. This analysis optimizes the selection algorithm that enables the identification of the best model by carefully following the nuances of the application at hand [3].

As the field of CKD prediction expands, the interaction between machine learning and clinical knowledge is an important factor. Collaboration between machine learning and medical professionals has created a link that combines algorithmic resources with clinical insights [4]. This integration facilitates the development of predictive models by infusing them with clinical knowledge, revealing key variables and hidden concepts. By combining the power of data-driven algorithms with the knowledge of medical professionals, our research seeks to create predictions that are not only effective but also dig deeper into the complexity of CKD diagnosis and prognosis in [5]. In this evolution, the integration of disparate data plays a crucial part in improving the accuracy of CKD prediction from a machine learning perspective [6]. The integration of these disparate data helps to create a holistic patient profile that provides a comprehensive view of the infection. Our research work highlights the importance of this diverse approach when navigating large volumes of data by examining its complexity to weed out meaningful patterns [7]. By reconciling these differences, our hope is not just to improve the accuracy of the prediction model; we strive to tailor treatments to the nuanced needs of patients.

Therefore, our journey to develop machine learning-driven clinical strategies for CKD management heralds a new era of personalized care [8]. As our research efforts build upon these good insights, they begin their journey of increased use. By exploring many different data lists and selection processes, we try to bring the accuracy of the prediction model to a new level [9]. Our aim is not to determine the prognosis of CKD, but to provide it with greater certainty. This effort does not only have the ability to enhance prognosis and early detection, but also leads to changes in patient outcomes [10]. Careful coordination of several machine learning techniques is necessary when exploring the CKD prediction area. Selecting the most appropriate algorithm is a dynamic process determined by factors such as the characteristics of the data, the components it contains, and the required levels [11]. These complex processes often require the deployment of a set of algorithms, each rigorously tested against consistent data. The result is a clear choice of algorithms; a design that adapts well to the complexity of its clinical application [12].

Important results from previous studies have pushed CKD prognostic research into unknown territory. The most important of these is the power of gradient amplification, which attracts attention due to its predictive power [13]. While DT [14] turned out to be important, the utility of KNNs in achieving accuracy in CKD prediction was clarified. Beyond the immediate impact, we hope that they can redefine the early diagnosis of CKD and ultimately improve patient outcomes. Inspired by these important insights, our research work is built on a solid foundation using various ML algorithms for CKD prediction [15]. The following sections describe the details of methodology adapted for prognosis of CKD using ensemble-based ML algorithms.

## 2. Methodology

**Figure 1:**
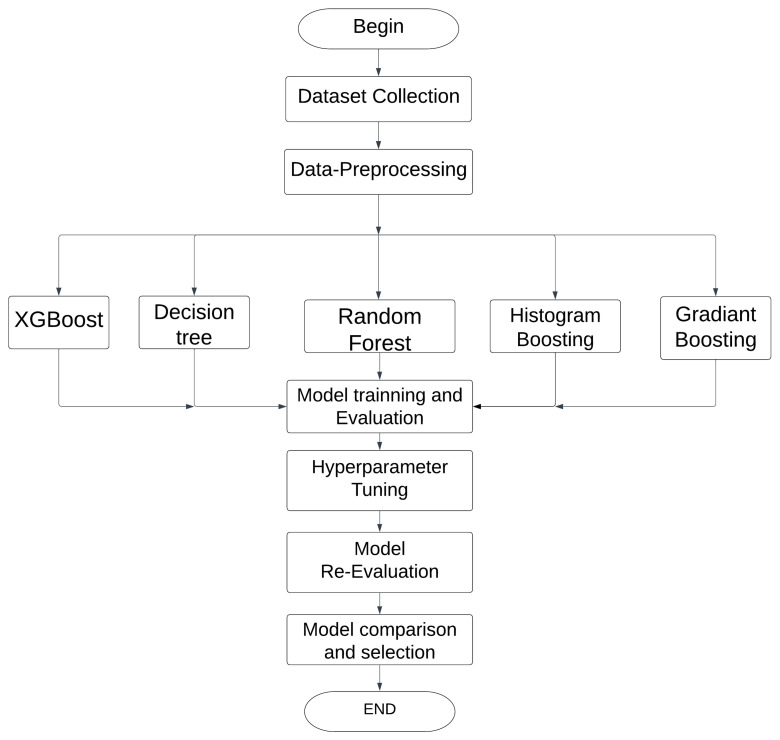
Workflow for comparative analysis of machine learning algorithms.

The methodology adapted for chronic kidney disease diagnosis is shown in Figure 1. This study involved collecting data on kidney disease patients, including clinical parameters and relevant features. Machine learning algorithms, such as gradient boost, decision tree, KNN, RF, HB, and XGB, were applied to the dataset. These algorithms were trained, evaluated, and used to analyze risk factors and associations related to kidney disease. The findings contribute to better prevention, early detection, and management strategies, eventually enhancing the health and quality of life of patients.

### 2.1. Data Preprocessing

The original data set [16] contained 11 numerical parameters, and the range of these parameters was different. Standard scaling was performed on the dataset using min-max normalization to bring dataset to the same range of [0, 1].

(1) $$X_{Normalize}=\frac{X-X_{\min}}{X_{\max}-X_{\min}}$$

The dataset also contained 13 categorical parameters. Label encoding was used for the categorical parameters in the dataset. This technique assigns a unique integer to each category. The split of the training and testing was 70–30; 70% of the dataset was used for training and 30% was used for testing.

### 2.2. K-Nearest Neighbor (KNN)

KNN is a flexible method used for regression and classification tasks. It makes predictions based on the similarity between fresh input data and labeled data points, locating the closest neighbors using a distance measure. The “lazy learner” KNN postpones computations until the moment of prediction. Considerations include choosing the appropriate “k” value and distance metric, as well as having sufficient labeled training data. It may be computationally inefficient for large datasets.

### 2.3. Parameters of KNN

KNN has key parameters including the number of neighbors (k), the distance metric, weighting scheme, and preprocessing techniques. These parameters influence the algorithm’s performance, accuracy, and handling of data. Optimal selection through experimentation and tuning is essential for achieving accurate results in classification or regression tasks.

Euclidean Distance: KNN algorithm does not involve a specific formula to estimate parameters or fit a model like parametric models such as linear regression or logistic regression. However, it uses a Euclidean formula to calculate the distance between the two points, illustrated by

(2) $$d=\sqrt{{\left(x_{2}-x_{1}\right)}^{2}+{\left(y_{2}-y_{1}\right)}^{2}}$$

The Euclidean distance formula is applied to KNN to determine the nearest neighbors for classification or regression by calculating the distances between the test data point and the training data points.

Inverse Weighted Distance: Equation (3)’s illustration of the inverse weighted distance approach shows that each neighbor’s weight is based on the inverse of their distance from the test site.

(3) $$Weight(i)=\frac{1}{distance(p,x_{i})}$$

**Figure 2:**
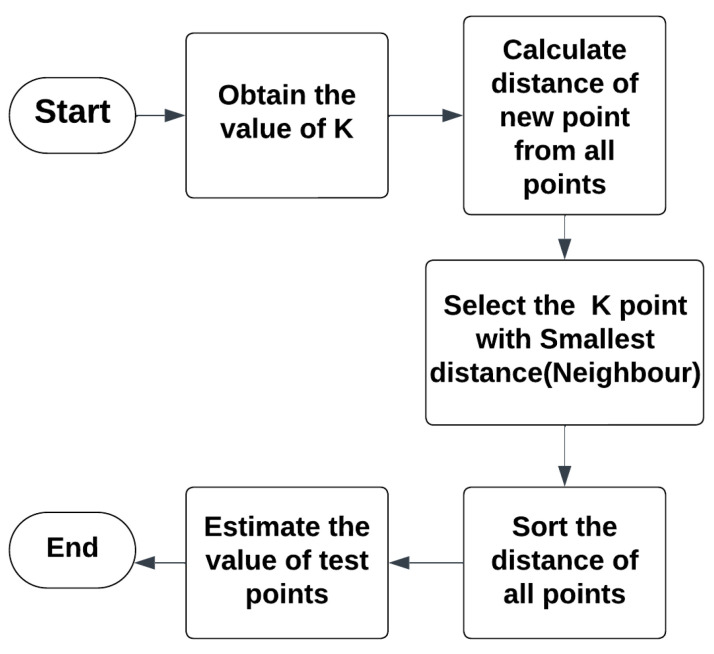
Flow chart of KNN.

The above equation represents the calculation of the weight (*Weight* (*i*)) for the *i^th^* neighbor, considering the distance between the test point *p* and the *i^th^* neighbor *xi*. Figure 2 shows the complete flow of the KNN algorithm.

### 2.4. Decision Tree (DT)

**Figure 3:**
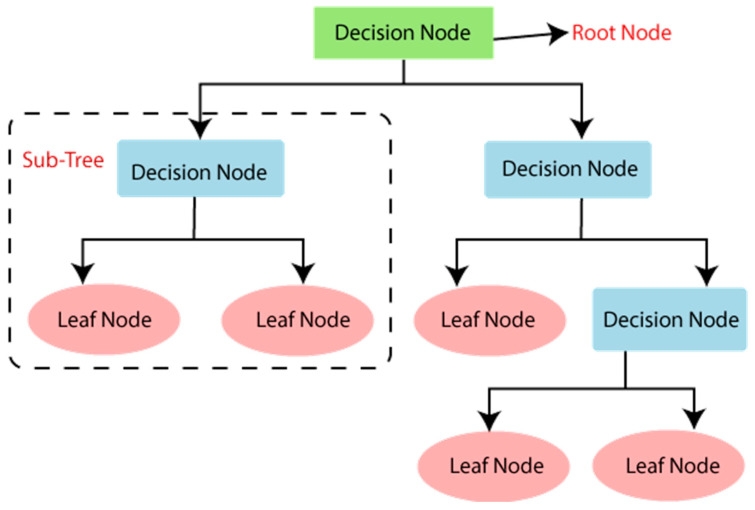
Workflow of decision tree.

DT is a supervised system that learns the set of rules that are used for every regression and categorization task. It has a shape like a flowchart, with each leaf node standing in for a final outcome or prediction, and each department standing in for a decision rule. The set of rules for the decision tree traverses the tree from the foundation node to a leaf node and makes decisions based solely on the values of the features. A decision rule is implemented at each inner node to select the department to monitor depending on the function value. The system continues until it reaches a leaf node, which provides the very last prediction or result. Decision trees are therefore susceptible to excessive fitting, when the model becomes very complex and catches noise in the data. Maximum tree depth settings and ensemble techniques like random forests are frequently used to reduce overfitting. Figure 3 shows how DT operates.

### 2.5. Parameters of DT

Gini Impurity: The Gini impurity quantifies the probability of incorrectly classifying a randomly chosen element in a database. The Gini impurity is represented by Equation (4), which calculates the impurity for a collection of class labels. It considers the probability of each class label p(c) in the set and sums the squared probabilities for all classes.

(4) $$Gini(p)=1-\sum p{\left(c\right)}^{2}$$

Entropy: Entropy is a measure of the level of disorder or impurity in a dataset. The entropy represented by Equation (5) calculates the entropy for a collection of class labels. It considers the probability of each class label p(c) in the set and sums the product of the probabilities and their logarithms.

(5) $$Entropy(p)=\sum p(c)\log_{2}p(c)$$

### 2.6. Random Forest (RF)

**Figure 4:**
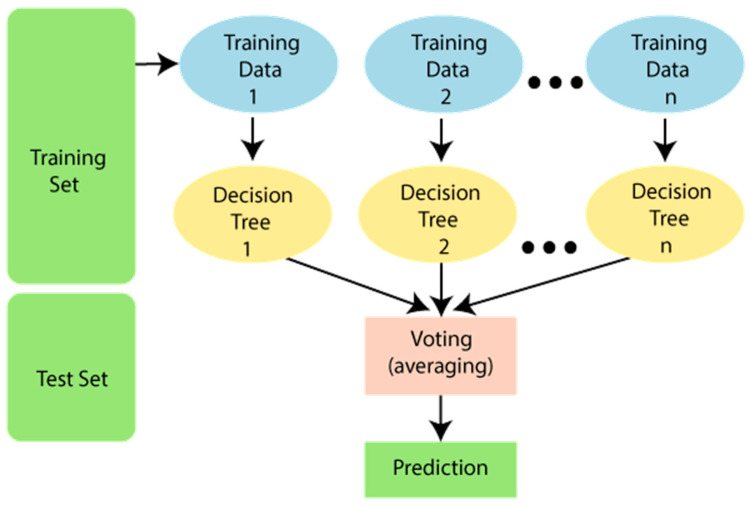
Workflow of random forest.

RF makes predictions that are more precise by combining the strengths of different decision trees. It is a method of collective learning that, by utilizing the idea of “wisdom of the crowd”, outperforms the individual predictions made by decision trees. RF has a number of benefits such as noise resistance, greater accuracy, important features, and versatility. The workflow of RF is shown in Figure 4.

### 2.7. Parameters of RF

The Gini Index: The Gini Index is the measure for evaluating the impurity of a node or a split in a random forest. It helps in selecting the feature to split on by assessing how pure or impure the resulting subsets would be. Mathematically, the Gini Index is calculated as the sum of the probabilities of each class label squared, subtracted from 1 as shown in Equation (6).

(6) $$Gini\;Index=1-\sum p{\left(c\right)}^{2}$$

Here, p(c) represents the probability of class c in the dataset or split.

Weighted Gini Index: Within the random forest algorithm, the impurity of a split at each node in the individual decision trees is determined using the weighted Gini Index. To produce predictions, this ensemble learning method mixes many decision trees. Equation (7) represents the random forest formula for the weighted Gini Index.

(7) $$Weighted\;Gini\;Index=\left(\sum \left(\frac{\left|D_{v}\right|}{\left|D\right|}\right)Index\times Gini\;Index\right)$$

Here, $\left|D_{v}\right|$ represents the number of occurrences in the subset $\left|D_{v}\right|$ after the split, |D| represents the total number of occurrences in the original dataset or node, and $Gini\;\left|D_{v}\right|$ represents the Gini Index of the subset $\left|D_{v}\right|$.

## 3. Boosting Algorithms

### 3.1. Gradient Boosting (GB)

Gradient boosting is an influential ensemble learning algorithm that merges weak predictive models, commonly decision trees, to forge a more potent and precise model. It harnesses the power of gradient descent to iteratively fine-tune the loss function after minimizing the gradients of the loss concerning the model’s predictions. This iterative process entails constructing weak learners to capture the residuals, progressively diminishing errors and augmenting the ensemble’s predictive capabilities. Employing regularization techniques effectively prevents overfitting, while hyperparameter tuning fine-tunes the model for optimal performance. Gradient boosting finds extensive applications across regression, classification, and ranking problems, with notable variations such as XGBoost and LightGBM elevating its efficiency and efficacy to remarkable levels. By combining the strengths of weak learners, gradient boosting has empowered data scientists to tackle complex objectives and achieve state-of-the-art results.

### 3.2. Histogram Boosting (HB)

Histogram-based gradient boosting is a technique used in gradient boosting algorithms, such as LightGBM and XGBoost. It involves the construction of histograms to efficiently compute the gradients and make predictions. This approach divides the feature space into discrete bins or buckets and constructs histograms based on these bins. It allows for faster computation and reduces the memory requirements compared to traditional gradient-boosting algorithms. Histogram gradient boosting has the advantage of reducing memory consumption and speeding up the training process, making it particularly useful for large-scale datasets. By using histograms, the algorithm can capture the distributional properties of the data and make efficient splits during tree construction.

### 3.3. Extreme Gradient Boosting (XGB)

XGB, which stands for extreme gradient boosting, is a cutting-edge machine learning technique that has earned global acclaim for its outstanding speed and efficacy in regression and classification applications. It employs gradient boosting, an ensemble learning strategy that combines numerous weak prediction models, often decision trees, to generate a stronger and more accurate overall model. XGBoost stands out by introducing several innovative features, such as regularization techniques, parallel tree construction, and advanced optimization algorithms, that enhance model accuracy and computational efficiency. This strong algorithm has demonstrated its worth in a variety of fields, including banking and healthcare, as well as computer vision and natural language processing. Because of its adaptability, speed, and excellent performance, XGBoost has become a top choice for researchers in data science and machine learning professionals looking for the best outcomes.

(8) $${\hat{y}}_{i}{}^{\left(t\right)}=\sum_{k=1}^{t}f_{k}(x_{i})={\hat{y}}_{i}{}^{\left(t-1\right)}+f_{t}(x_{i})$$

The XGboost algorithm integrates the prediction of week classifiers and achieves strong classification as shown in Equation (8), where ${\hat{y}}_{i}{}^{\left(t-1\right)}$ is the previously generated tree model and $f_{t}(x_{i})$ is the newly generated tree model.

### 3.4. Parameters of Boosting Algorithm

Learning Rate (η): The learning rate determines how much each base learner (e.g., tree) contributes to the final ensemble. It scales the influence of each learner’s prediction during the boosting phase. The formula for updating the ensemble’s predictions with the learning rate is shown in Equation (9)

(9) $$NP=P_{\mathrm{Pre}}+\eta L_{\mathrm{Pre}}$$

where NP is the new prediction, $P_{\mathrm{Pre}}$ is the previous prediction, and $L_{\mathrm{Pre}}$ is the learner prediction.

Residual Calculation: Boosting algorithms aim to minimize the residuals between the ensemble’s predictions and the true values. The residual for each instance in the training data is calculated by Equation (10)

(10) Residual=TrueValue−Ensemble Prediction

Weighted Sample Importance: Boosting algorithms assign weights to each instance in the training data to prioritize the importance of different samples. The weights are typically updated based on the errors or residuals of the ensemble’s predictions. The formula for calculating the updated sample weights is given by Equation (11)

(11) sample_weight=sample_weight*exp(−η*residual)

Base Learner Weight: Boosting algorithms assign weights to each base learner (e.g., tree) in the ensemble based on their performance. The formula for calculating the weight of a base learner is typically based on the algorithm-specific loss function and the importance of the learner’s predictions. The exact formula may change depending on the specific boosting algorithm used. The following section describes details of all the parameters and their impact for CKD.

## 4. Clinical Parameters

**Figure 5:**
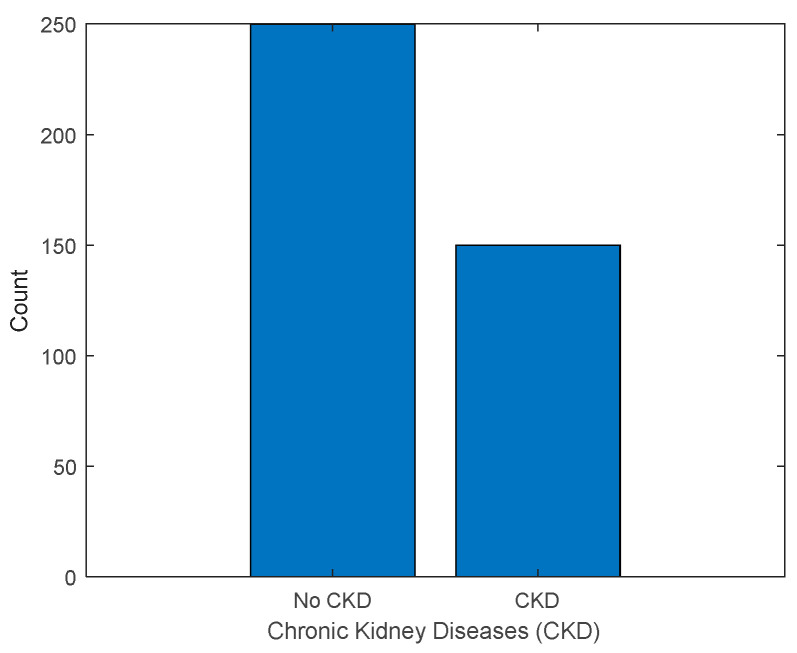
Count of number of patients with CKD and no CKD.

The factors causing renal disease are discussed in this section. By incorporating these clinical indicators alongside other features, researchers can thoroughly analyze the risk factors and associations associated with kidney disease. This comprehensive understanding significantly contributes to the advancement of prevention, early detection, and management strategies for kidney disease. From the survey, it was observed that proteinuria is an early predictor of functional and survival outcomes after renal surgery [17,18,19]. For the reference study, the dataset was collected from Kaggle [16]. In total, 400 patients’ data were used, with 250 patients not having chronic kidney diseases and 150 patients are diagnosed with chronic kidney diseases. Figure 5 shows the count.

A total of 24 parameters are used for the diagnosis of CKD. Of these 24, 11 parameters have values in the numerical range while 13 features have categorical values. Age, blood pressure, specific gravity, albumin, sugar red blood cells, pus cells, pus cell clusters, bacteria, blood urea, serum creatinine, sodium, potassium, hemoglobin, packed cell volume, white blood cell count, red blood count, hypertension, diabetes mellitus, coronary artery disease, appetite, edema, and anemia are some of these characteristics. Table 1 displays the numerical statistics together with their minimum, maximum, and mean values.

Categorical features are shown in Table 2. Specific gravity gives a gravity range from 1.005 to 1.025. Albumin and sugar are leveled from 0 to 5. Both pus cells and red blood cells can be classified as normal or pathological. Bacteria and pus cell clumps are classified as present or absent. Anemia, pedal edema, coronary artery disease, hypertension, and diabetes mellitus are categorized as either yes or no.

Table 3 depicts the average values of parameters that lead to CKD and those which do not. From Table 3, we can observe how these features or parameters relate to CKD.

The average age of people who do not have CKD is 46, whereas it increases to 54 for people with CKD. Normal average blood pressure value is around 70 mm/Hg, whereas it increases to 79 mm/Hg for people with CKD. Normal average blood glucose random is 107 mgs/dL, while it increases to 175 mgs/dL for people with CKD. Normal average blood urea level is 32.79 mgs/dL, which increases to 72.38 mgs/dL for people with CKD. Serum creatinine level is 0.8689 mgs/dL in normal people, which increases to 4.41 mgs/dL for people with CKD.

Average sodium level for patients with no CKD is observed to be 141.73 mEq/L, whereas it decreases to 133.9 mEq/L for patients with CKD. Average potassium level for patients with no CKD is observed to be 4.33 mEq/L, whereas for patients with CKD, it is 4.87 mEq/L. Average hemoglobin level for patients with no CKD is found to be 15.15 gms, whereas it decreases to 10.64 gms for patients with CKD. Average value of packed cell volume for patients without CKD is 46.33, which decreases to 32.93 for patients with CKD. Similarly, average white blood cell count for patients without CKD is 7705.59 cells/cumm, which increases to 9069 cells/cumm for patients with CKD. Lastly, the average red blood cell count for patients without CKD is 5.37 million/cmm, which decreases to 3.94 million/cmm for patients with CKD. These parameters are described below.

### 4.1. Age

The age of the patient is an important factor in evaluating kidney disease. Kidney function naturally declines with age, and older individuals are more susceptible to kidney disease. Renal structural and functional changes brought on by aging can influence the onset and progression of renal disease. Assessing age helps identify individuals who may be at higher risk or require specific considerations for kidney disease management.

### 4.2. High Blood Pressure

A leading factor in kidney disease is high blood pressure, commonly referred to as hypertension. The kidneys’ ability to operate can be harmed by long-term hypertension, which can harm their blood arteries. We determine the relationship between blood pressure and renal disease by examining blood pressure levels. To stop or decrease the course of kidney disease among individuals with high blood pressure, diligent monitoring and management is necessary.

**Figure 6:**
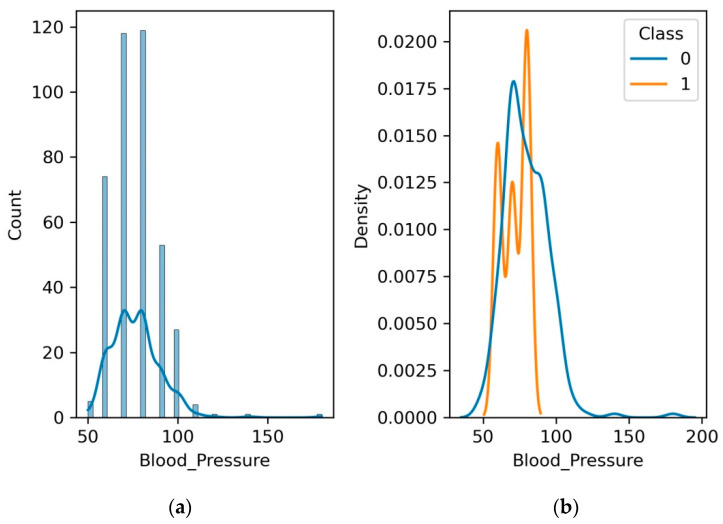
(**a**) Histogram and PDF plot for blood pressure; (**b**) PDF plot for the range of blood pressure level for patients with CKD and no CKD.

Figure 6 underscores the clinical significance of blood pressure as a potential marker of kidney health. Observing a concurrence of elevated blood pressure and escalating kidney disease severity may serve as an early indicator, prompting vigilant evaluation of renal function. Conversely, maintaining optimal blood pressure levels might contribute to preserving kidney well-being. We encourage researchers and healthcare professionals to unravel the mechanisms that underlie this relationship, potentially unraveling novel avenues for early detection and personalized intervention strategies. It can be observed in Figure 6b that the range of blood pressure for non-CKD patients is nearly 40 to 120, while it is 50 to 90 for patients with CKD.

### 4.3. Blood Glucose Random

Blood glucose is the sugar in the blood that gives you energy. It is important for kidney function. When blood glucose levels are too high, it can damage the kidneys. This is because high blood glucose levels can cause the kidneys to filter out too much protein. The protein then builds up in the kidneys and can damage them. Kidney failure is a dangerous condition that can result from kidney disease. Sustaining a healthy blood glucose level is crucial when suffering from renal disease. This can reduce the progression of renal disease and stop kidney failure.

**Figure 7:**
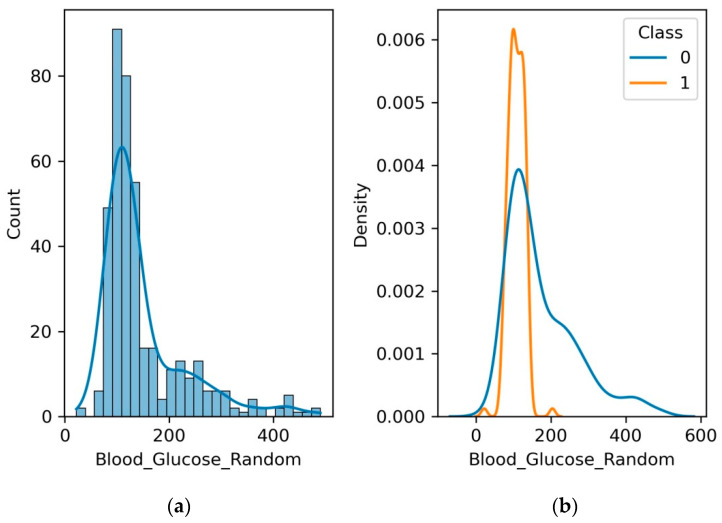
(**a**) Histogram and PDF plot for blood glucose level; (**b**) PDF plot for the range of blood glucose level for patients with CKD and no CKD.

Figure 7 depicts the clinical significance of blood glucose as a potential contributor to renal health. Fluctuations in blood glucose levels can exert a profound impact on kidney function over time. Monitoring blood glucose trends provides healthcare professionals with insights into potential kidney-related implications, driving timely assessment and informed management. We encourage researchers and medical professionals to explore the intricate mechanisms that underlie this relationship, potentially revealing novel insights that could reshape our understanding of kidney health assessments and therapeutic strategies. It is observed from Figure 7b that the range of random blood glucose level is limited to 50 to 150 in patients with no CKD, whereas it increases to 0 to 600 for patients with CKD.

### 4.4. Blood Urea Nitrogen

Another crucial clinical test that evaluates kidney function is blood urea nitrogen (BUN). BUN gauges the blood’s nitrogen content, which comes from the waste product urea. Increased BUN levels may signal poor renal and waste clearance. Monitoring BUN levels aids in determining how well the kidneys function in filtering and eliminating waste.

**Figure 8:**
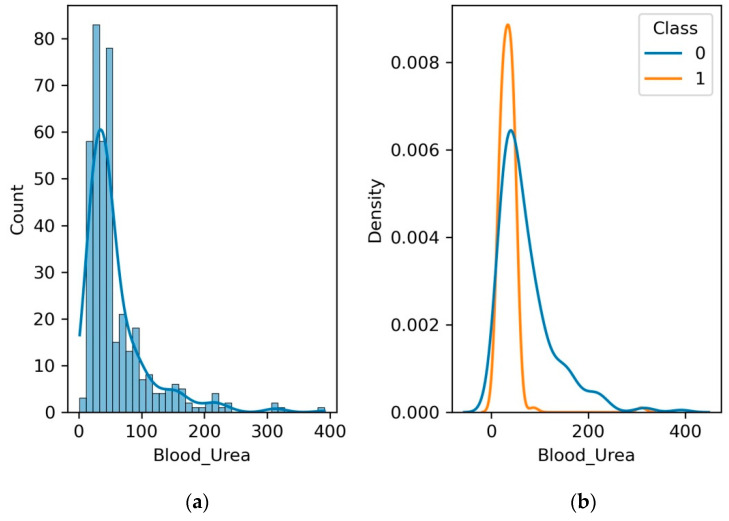
(**a**) Histogram and PDF plot for blood urea; (**b**) PDF plot for the range of blood urea level for patients with CKD and no CKD.

Figure 8 depicts the clinical significance of BUN as a potential indicator of renal health. BUN serves as a marker of how effectively the kidneys are filtering and excreting waste products from the blood. Observing fluctuations in BUN levels can offer healthcare practitioners crucial insights into kidney function, prompting further assessment and proactive management. The depiction of blood urea nitrogen as an indicator of potential renal challenges highlights its pivotal place in healthcare diagnostics. It can observed from Figure 8b that the normal range of BU is 0 to 400, which decreases to 100 for patients with CKD.

### 4.5. Serum Creatinine

A crucial clinical indicator used to evaluate kidney function is serum creatinine. It is a waste product produced by muscle metabolism that the kidneys filter out of the blood. Elevated serum creatinine levels signify deteriorated kidney health and can be used as an indicator of kidney disease. Regular monitoring of serum creatinine levels helps evaluate the progression and severity of kidney disease.

**Figure 9:**
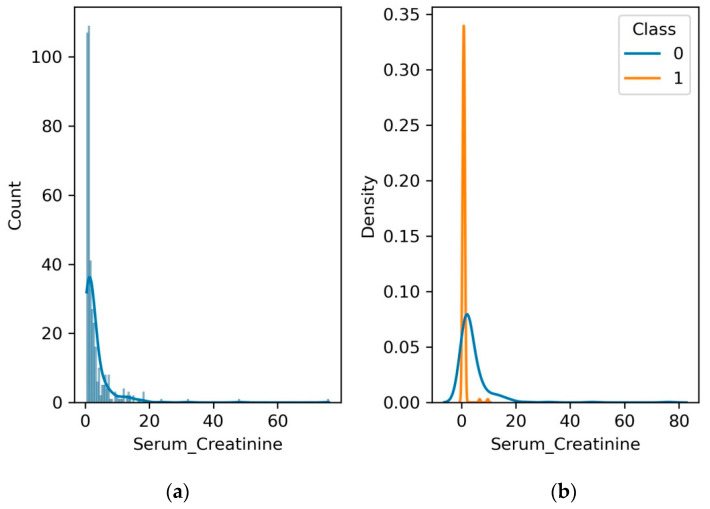
(**a**) Histogram and PDF plot for serum creatinine; (**b**) PDF plot for the range of serum creatinine level for patients with CKD and no CKD.

Figure 9 shows the clinical significance of serum creatinine as a potential indicator of renal health. Serum creatinine is a modifiable risk factor for kidney disease, and it can be used to monitor the progression of kidney disease and to assess the effectiveness of treatment, encouraging us to venture deeper into the complex interplay between serum creatinine and renal health. We invite researchers and medical professionals to delve into the underlying mechanisms that drive this relationship, potentially unearthing novel insights that could shape the landscape of kidney health assessments and interventions. It can be observed from Figure 9a that density function and count for the serum creatinine level is in the range of 0–20. Figure 9b shows that the normal serum creatinine level is 0 to 20, which decreases to 5 for patients with CKD.

### 4.6. Serum Sodium

**Figure 10:**
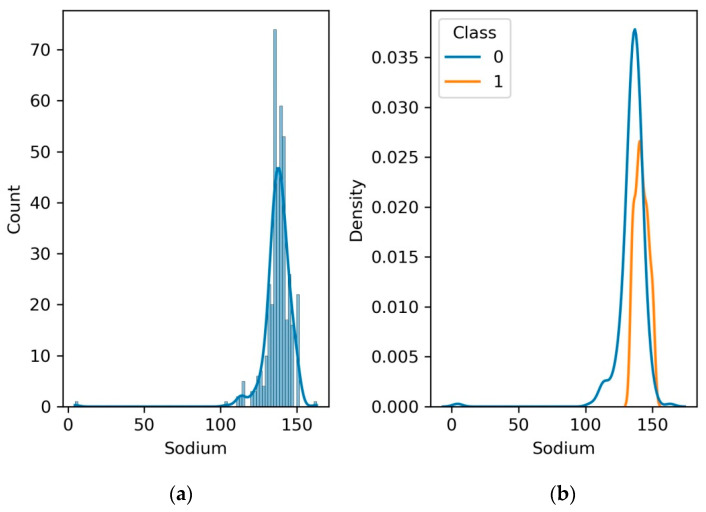
(**a**) Histogram and PDF plot for sodium; (**b**) PDF plot for the range of sodium level for patients with CKD and no CKD.

Serum sodium levels are an essential clinical measurement that reflects the balance of water and electrolytes in the body. Abnormal sodium levels can indicate kidney dysfunction and electrolyte imbalances. The kidneys are essential for sustaining sodium balance, and impaired kidney function can lead to abnormal sodium levels. Monitoring serum sodium helps identify electrolyte imbalances associated with kidney disease. Figure 10a shows that density function and count for sodium level are in the range of 100–150. Figure 10b depicts that the normal serum sodium level is 100 to 150, whereas for patients with CKD it is 100–150.

### 4.7. Serum Potassium

Serum potassium levels are critical for maintaining proper kidney function. The kidneys help regulate potassium levels in the body. Abnormal potassium levels can be an indication of kidney disease. Decreased kidney function can lead to an accumulation of potassium, resulting in hyperkalemia. Monitoring serum potassium levels helps assess kidney health and the risk of electrolyte imbalances.

**Figure 11:**
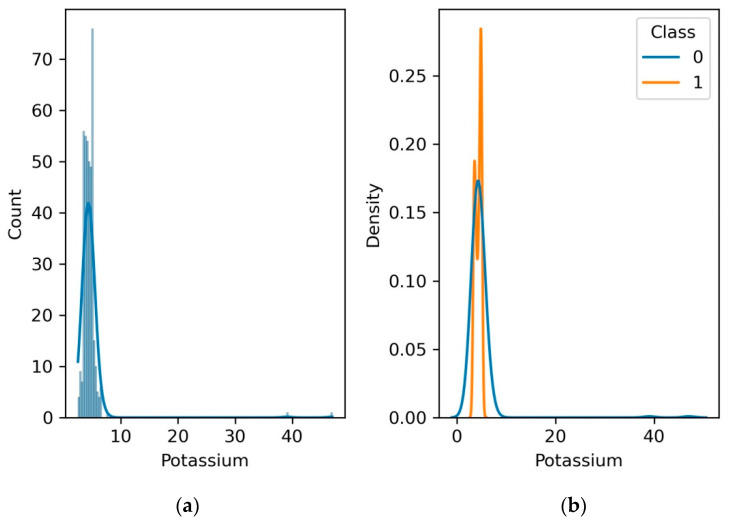
(**a**) Histogram and PDF plot for potassium; (**b**) PDF plot for the range of potassium level for patients with CKD and no CKD.

Figure 11 shows the clinical significance of potassium as a potential indicator of renal health. The interplay between potassium levels and kidney function offers a glimpse into the delicate balance maintained within the body. Monitoring shifts in potassium concentrations may provide early cues for potential kidney dysfunction, guiding healthcare practitioners toward timely intervention. We invite researchers and medical professionals to unravel the underlying mechanisms that drive this relationship, potentially unveiling novel insights that could revolutionize our understanding of kidney health management.

### 4.8. Hemoglobin

Hemoglobin’s relationship with kidney function is profound. Erythropoietin, a hormone that promotes the synthesis of red blood cells and thus affects hemoglobin levels, is produced mostly by the kidneys. Elevated hemoglobin levels can signify a harmonious interplay between the kidneys and erythropoietic processes, indicating optimal renal function. Hemoglobin emerges as a dynamic ally, offering a comprehensive insight into the intricate interplay between renal function and overall well-being. This multifaceted protein extends its influence beyond its renowned role in oxygen transport, serving as a sentinel of kidney health and a potent marker of potential kidney disease.

**Figure 12:**
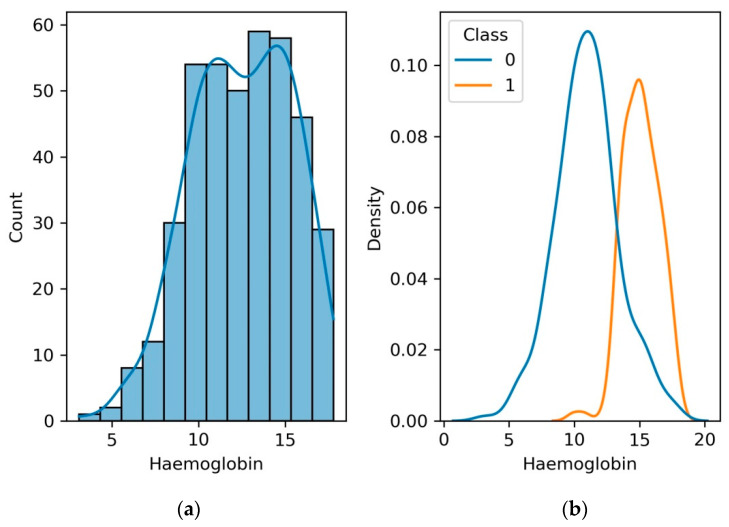
(**a**) Histogram and PDF plot for hemoglobin; (**b**) PDF plot for the range of hemoglobin level for patients with CKD and no CKD.

Figure 12 shows a discernible pattern emerges as the data points converge and diverge along the axes. Higher hemoglobin levels, indicative of enhanced oxygen-carrying capacity, appear to align with healthier kidney function, manifested through lower kidney disease severity. Conversely, lower hemoglobin levels seem to coincide with an escalation in kidney disease, portraying a potential link between reduced oxygen-carrying capacity and deteriorating renal health. The graph underscores the clinical significance of hemoglobin as a potential indicator of kidney disease presence and progression. A downward trend in hemoglobin levels might warrant a vigilant assessment for kidney dysfunction, while maintaining optimal hemoglobin concentrations could signify improved renal health.

By incorporating these clinical measurements, researchers can conduct a thorough analysis of the risk factors and associations associated with kidney disease. This comprehensive understanding significantly contributes to the advancement of prevention, early detection, and management strategies for kidney disease, ultimately leading to improved health results and a higher quality of life.

### 4.9. Correlation Matrix

**Figure 13:**
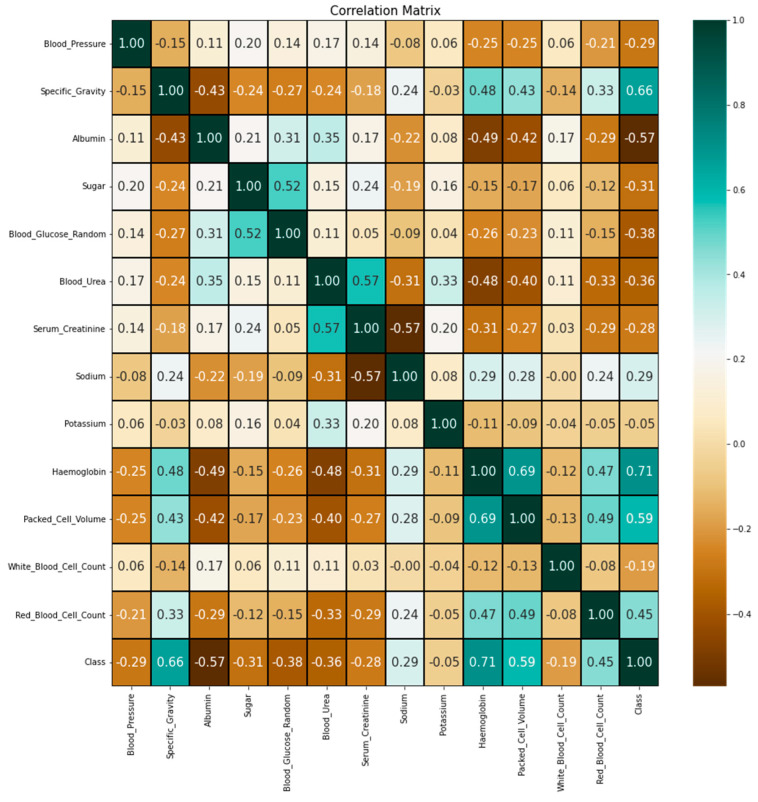
Correlation matrix between the parameters and output class.

A correlation matrix is a key tool in statistics and machine learning for understanding the relation between different variables. It provides a numerical representation of the strength and direction of linear relationships between pairs of variables. In the context of machine learning, a correlation matrix is often used for feature selection, data preprocessing, and exploring the data’s underlying patterns. The correlation coefficient for the input features and target output is calculated using

(12) $$r=\frac{\sum_{i=1}^{N}\left(x_{i}-\bar{x}\right)\left(y_{i}-\bar{y}\right)}{\sqrt{\sum_{i=1}^{N}{\left(x_{i}-\bar{x}\right)}^{2}\sum_{i=1}^{N}{\left(y_{i}-\bar{y}\right)}^{2}}}$$

where $x_{i}$ is the $i^{th}$ data sample of the input features, $y_{i}$ is the $i^{th}$ data sample of the output, and N is the total number of data samples. The correlation matrix calculated for features with numerical values with output is shown in Figure 13.

Typically, the correlation matrix values fall between −1 and 1. Strong negative correlation is shown by a correlation value of −1, which means that as one variable rises, the other falls. When showing a high positive connection, a correlation value of 1 implies that as one variable rises, the other rises as well. A correlation coefficient that is around zero indicates that there is little to no linear relationship between the variables. How close the value is to −1 or 1 determines how strong the correlation is. The association is stronger the closer the value is to these extremes.

## 5. Results

In this section, we investigate the usefulness of various machine learning algorithms for detecting and predicting renal disease, as well as show the experimental findings from our study. We tested a number of techniques, including gradient boost (GB), decision tree (DT), K-nearest neighbors (KNN), random forest (RF), histogram boosting (HB), and XGBoost. Quantitative assessment of these ML algorithms was carried out using various parameters which are described below.

### Confusion Matrix

Shown below is a N × N confusion matrix, where N is the number of target classes. We compared the goal values to those predicted by the ML model in this matrix. Confusion matrix for the classification of chronic kidney diseases is shown in Table 4.

Using TP, FN, FP, and TN, various quantitative parameters are calculated.

Accuracy is the percentage of accurate positive predictions among all positive predictions, which demonstrates the model’s capacity to recognize positive cases, calculated by $Accuracy=\frac{TP+TN}{N}$.

Precision is calculated as the percentage of correct positive predictions among all positive predictions, and it indicates how well the model can identify positive cases. $Precision=\frac{TP}{TP+FP}$.

Recall is computed as the proportion of genuine positive predictions out of all real positive instances, indicating the model’s capacity to catch positive cases, calculated by $Recall=\frac{TP}{TP+FN}$.

$F_{1}\_Score$ is the harmonic mean of precision and recall gives us a fair comparison of the two measurements.

(13) $$F_{1}\_Score=2\times \frac{Precision\times Recall}{Precision+Recall}$$

Jaccard similarity is the measure of similarity between two sets. For example, if y is actual label and $\hat{y}$ is predicted label, it is calculated using

(14) $$JS(y,\hat{y})=\frac{\left|y\cap \hat{y}\right|}{\left|y\right|+\left|\hat{y}\right|-\left|y\cap \hat{y}\right|}$$

Log loss is a loss function used to assess the effectiveness of probabilistic classification models. It is also referred to as logarithmic loss or cross-entropy loss.

(15) $$logloss=-\frac{1}{N}\sum_{i=1}^{N}y_{i}\log\left(p({\hat{y}}_{i})\right)+\left(1-y_{i}\right)\log\left(1-p({\hat{y}}_{i})\right)$$

AUC ROC Curves: The performance of a binary classification model is visually represented by ROC, which illustrates the relationship between true positive rate and false positive rate.

(16) $$TPR=\frac{TP}{TP+FN}\;\;\;\;\;and\;\;\;\;\;\;FPR=\frac{FP}{FP+TN}$$

The quantitative assessment of six ML algorithms using the above parameters performed on the testing dataset are shown in Table 5. From Table 5, it can be observed from the outcome that random forest (RF) achieved the highest accuracy of 98.75%, followed by histogram boosting (HB) and XGBoost (XGB) with 98.75% each, gradient boosting (GB) with 97.50%, decision tree (DT) with 97.50%, and K-nearest neighbors (KNN) with 96.25%.

**Figure 14:**
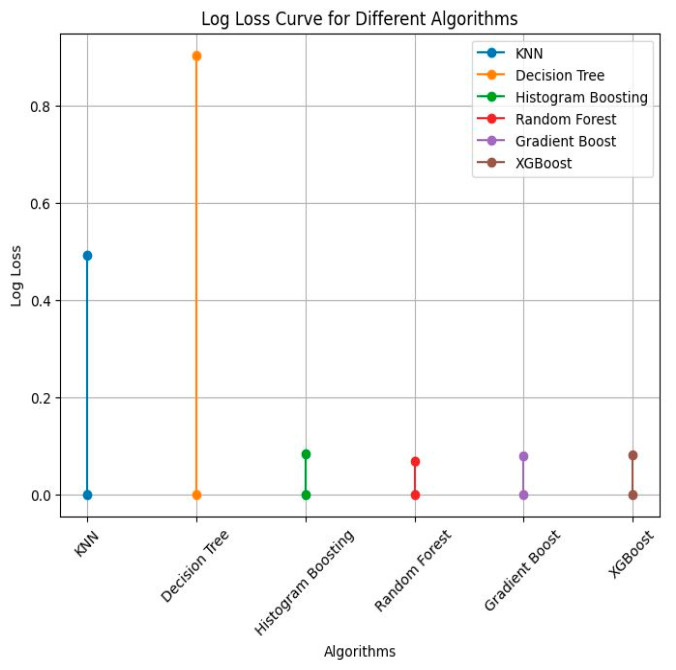
Log loss obtained for six ML algorithms.

The Jaccard similarity scores varied among the models, with RF, HB, and XGB reporting the highest similarity of 96.42%, followed by GB with 96.42%, KNN with 93.10%, and DT with 90.00%. When considering precision, DT achieved the highest value of 98.00%, followed by GB, RF, HB, XGB, and KNN with 97.00%, 98.00%, 97.00%, 98.00%, and 60.00%, respectively. For recall, GB, DT, RF, HB, XGB, and KNN obtained values of 98.00%, 96.96%, 96.00%, 1.00%, 1.00%, and 62.00%, respectively. The F1 scores show that DT achieved the highest value of 98.00%, followed by GB with 96.00%, and RF, HB, XGB, and KNN with 98.00%, 98.00%, 98.00%, and 61.00%, respectively. Log loss values collected for these six ML algorithms are shown in Figure 14.

**Figure 15:**
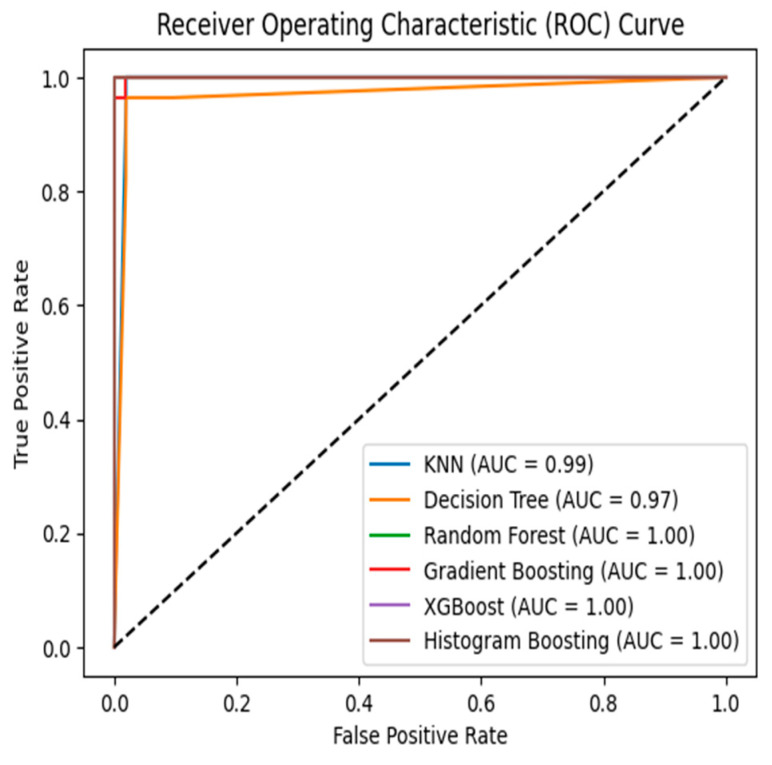
ROC curve obtained for six ML algorithms.

Regarding log loss, RF achieved the lowest value of 0.048, followed by XGB with 0.036, GB with 0.073, HB with 0.030, DT with 1.351, and KNN with 0.485. These results indicate that RF performed exceptionally well in terms of accuracy and log loss, while DT showed high precision and F1 score. Each model has its strengths and weaknesses in different evaluation metrics. The ROC AUC curve plotted for these six ML algorithms is shown in Figure 15. This shows that the model is biased with given data and may be sensitive to new data. This can be improved by hyperparameter tuning. Our experimental study shows that DT, RF, GB, and XGB show an equivalent performance in accuracy, precision, recall, F1 score, and AUC-ROC. This shows that models are not biased towards class but biased towards data. By selecting the optimum number of trees, features, gain impurity, minimum samples in leaf node, and minimum samples for splitting node and resampling, model generalization is possible [20,21,22].

## 6. Discussion

In this research paper, we investigated the performance of six different machine learning algorithms for the prediction of kidney disease. The algorithms used were K-nearest neighbors (KNN), decision tree (DT), histogram-based gradient boosting (HB), random forest (RF), gradient boosting (GB), and extreme gradient boosting (XGB). Our objective was to compare the predictive capabilities of these algorithms and identify the most reliable and precise way for predicting kidney disease.

Performance Comparison: We evaluated the performance of each algorithm using a comprehensive set of evaluation metrics, including accuracy, precision, recall, F1 score, and area under the receiver operating characteristic curve (AUC-ROC). The results demonstrated varying levels of performance across the algorithms, indicating differences in their ability to correctly predict the presence or absence of kidney disease. The results show that overall, AUC-ROC was significantly good and indicated the best performance in predictions of either case, but this is not the single parameter to make an overall decision about model quality with. Model quality can be judged with effective performance of F1 score, accuracy, recall, and precision. All models, except for KNN and HB, showed significant results for all parameters. KNN and HB showed higher accuracy but precision, recall and F1 score were on the compromising side, indicating that the performance of applied algorithms, i.e., KNN and HB, is biased towards one class.

Accuracy and F1 Score: Among the algorithms, XGB achieved the highest accuracy and F1 score, indicating its overall superiority in classifying instances correctly and maintaining a fine balance between recall and precision. The RF and GB algorithms also performed well, showing competitive accuracy and F1 scores. These findings are consistent with the existing literature on the strengths of ensemble methods like RF and GB for complex classification tasks.

Precision and Recall: Although XGB demonstrated high accuracy and F1 score, it is essential to consider the recall and precision values in the context of kidney disease prediction. RF exhibited higher precision compared to the other algorithms, signifying its proficiency in correctly identifying true positive cases of kidney disease. Conversely, KNN showed higher recall values, implying its ability to capture a greater proportion of actual positive cases, but at the expense of precision.

AUC-ROC: The AUC-ROC metric provides a measure of the algorithms’ ability to differentiate between positive and negative instances. XGB and RF attained the highest AUC-ROC scores, indicating their strong discriminative power. On the other hand, DT and KNN presented comparatively lower AUC-ROC scores, suggesting a reduced ability to distinguish between the two classes effectively.

Computational Efficiency: In addition to predictive performance, we also considered the computational efficiency of the algorithms. KNN and DT are relatively simple algorithms with lower computational demands, while RF, GB, and XGB require more computational resources due to their ensemble nature and iterative training process. However, the trade-off between accuracy and computational efficiency should be considered when choosing an appropriate algorithm for kidney disease prediction, especially in resource-constrained environments.

Interpretability: Another important aspect to consider is the interpretability of the models. DT and KNN are more interpretable, as they produce decision rules and exemplify instance-based reasoning, respectively. In contrast, ensemble methods like RF, GB, and XGB are more challenging to interpret due to their ensemble nature and the aggregation of multiple weak learners. Interpretability may be crucial in certain clinical applications where it is essential to understand the decision-making process of model.

Our study provides a comprehensive evaluation of six different machine learning algorithms for kidney disease prediction. While XGB demonstrated the highest overall predictive performance, RF and GB also exhibited strong capabilities. Depending on the application’s requirements, researchers and practitioners may choose the appropriate algorithm based on a trade-off between accuracy, precision, recall, AUC-ROC, computational efficiency, and interpretability.

## 7. Conclusions

Finally, this study investigated the efficacy of various machine learning algorithms for renal disease diagnosis and prognosis. Gradient boost, decision tree, KNN, random forest, histogram boosting, and XGBoost were thoroughly assessed for their distinct capabilities and contributions to the challenge. The outcomes were encouraging, with all algorithms displaying excellent performance. Gradient boost, XGBoost, and histogram boosting demonstrated good accuracy and an exceptional capacity to grasp complex correlations in the dataset. These models show a lot of potential for making predictions with high accuracy in real-world applications. By providing details about the process of decision-making and highlighting crucial elements influencing prediction, random forest and decision trees provided significant interpretability. Meanwhile, KNN excelled at capturing local patterns, making it a powerful tool for situations requiring localized insights.

Several important predictors were identified by the feature importance analysis across several algorithms, including serum creatinine level, blood pressure, and age. These discoveries highlight the clinical importance of these parameters in the diagnosis and prognosis of renal disease. Early detection of kidney illness is critical for timely intervention and effective care, and the predictors revealed can serve as critical markers for medical practitioners. While this study returned promising outcomes, there is still opportunity for progress in the sector. Future research efforts could concentrate on improving the accuracy and resilience of existing algorithms. Furthermore, investigating ensemble methods that combine the characteristics of various algorithms and incorporate additional important aspects may result in even more precise and dependable predictions.

In conclusion, the thorough study of machine learning algorithms in renal disease diagnosis adds new insights to the field of healthcare. Medical professionals can make informed judgments and deliver individualized treatment strategies by using the power of these algorithms, ultimately leading to enhanced patient outcomes and overall kidney health. The search for early detection, intervention, and successful management of kidney disease continues, and machine learning is a valuable tool in that pursuit.

## Figures and Tables

**Table 1:** Features with numerical values.

SN	Parameter or Feature	Min Value	Maximum Value	Mean Value
1	Age in years	2	90	51.48
2	Blood pressure in mm/Hg	50	180	76.46
3	Blood glucose random in mgs/dL	22	490	148.03
4	Blood urea in mgs/dL	1.50	391.0	57.42
5	Serum creatinine in mgs/dL	0.40	76.00	3.07
6	Sodium in mEq/L	4.50	163.0	137.52
7	Potassium in mEq/L	2.50	47.00	4.62
8	Hemoglobin in gms	3.10	17.80	12.52
9	Packed cell volume	9	54	48.32
10	White blood cell count in cells/cumm	2200	26,400	7658.82
11	Red blood cell count in millions/cmm	2.1	8	5.51

**Table 2:** Features with categories.

SN	Parameter or Feature	Value or Attribute
1	Specific Gravity	1.005, 1.010, 1.015, 1.020, 1.025
2	Albumin	0, 1, 2, 3, 4, 5
3	Sugar	0, 1, 2, 3, 4, 5
4	Red Blood Cells	Normal or abnormal
5	Pus Cell	Normal or abnormal
6	Pus Cell Clump	Present or not present
7	Bacteria	Present or not present
8	Hypertension	Yes or No
9	Diabetes Mellitus	Yes or No
10	Coronary Artery Disease	Yes or No
11	Appetite	Good or Poor
12	Pedal Edema	Yes or No
13	Anemia	Yes or No

**Table 3:** Average values of various parameters for classification of chronic kidney diseases.

SN	Parameter or Feature	No Chronic Kidney Diseases	Chronic Kidney Diseases
1	Age	46	54
2	Blood Pressure	71	79
3	Blood Glucose Random	107	175
4	Blood Urea	32.79	72.38
5	Serum Creatinine	0.868966	4.41
6	Sodium	141.73	133.90
7	Potassium	4.33	4.87
8	Hemoglobin	15.18	10.647
9	Packed Cell Volume	46.33	32.93
10	White Blood Cell Count	7705.59	9069.53
11	Red Blood Cell Count	5.37	3.94

**Table 4:** Confusion matrix for classification of chronic kidney diseases.

		Predicted Class	
Actual Class		Chronic Kidney Disease	Non-Chronic Kidney Disease
	Chronic Kidney Disease	True Positive (TP)	False Negative (FN)
	Non-Chronic Kidney Disease	False Positive (FP)	True Negative (TN)

**Table 5:** Performance analysis of six ML algorithms using various quantitative parameters.

	Results in % Using Six ML Algorithms					
Parameters	KNN	DT	HB	RF	GB	XGB
Accuracy	98.75	97.50	98.75	98.75	97.50	98.75
Jaccard Similarity	93.10	90.00	96.42	96.42	96.42	96.42
Precision	66.00	97.00	60.00	97.00	97.00	99.00
Recall	66.00	97.00	62.00	97.00	97.00	99.00
F1 Score	66.00	97.00	61.00	98.00	97.00	99.00
ROC AUC	99.00	97.00	100.00	100.00	100.00	100.00

## Data Availability

No new data were created or analyzed in this study. The data is publicly available on https://doi.org/10.24432/C5G020.
